# Early impoverished environment delays the maturation of cerebral cortex

**DOI:** 10.1038/s41598-018-19459-y

**Published:** 2018-01-19

**Authors:** Roberta Narducci, Laura Baroncelli, Gabriele Sansevero, Tatjana Begenisic, Concetta Prontera, Alessandro Sale, Maria Cristina Cenni, Nicoletta Berardi, Lamberto Maffei

**Affiliations:** 1 Institute of Neuroscience, National Research Council (CNR), Via Moruzzi 1, I-56124 Pisa, Italy; 2 Department of Neuroscience, Psychology, Drug Research and Child Health NEUROFARBA, University of Florence, Area San Salvi – Pad. 26, I-50135 Florence, Italy; 3 Fondazione G. Monasterio CNR-Regione Toscana, via Moruzzi 1, I-56124 Pisa, Italy

**Keywords:** Neuronal development, Neural circuits

## Abstract

The influence of exposure to impoverished environments on brain development is unexplored since most studies investigated how environmental impoverishment affects adult brain. To shed light on the impact of early impoverishment on developmental trajectories of the nervous system, we developed a protocol of environmental impoverishment in which dams and pups lived from birth in a condition of reduced sensory-motor stimulation. Focusing on visual system, we measured two indexes of functional development, that is visual acuity, assessed by using Visual Evoked Potentials (VEPs), and VEP latency. In addition, we assessed in the visual cortex levels of Insulin-Like Growth Factor 1 (IGF-1) and myelin maturation, together with the expression of the GABA biosynthetic enzyme GAD67. We found that early impoverishment strongly delays visual acuity and VEP latency development. These functional changes were accompanied by a significant reduction of IGF-1 protein and GAD67 expression, as well as by delayed myelination of nerve fibers, in the visual cortex of impoverished pups. Thus, exposure to impoverished living conditions causes a significant alteration of developmental trajectories leading to a prominent delay of brain maturation. These results underscore the significance of adequate levels of environmental stimulation for the maturation of central nervous system.

## Introduction

The complexity of early stimulation can modulate brain developmental trajectories and can induce long-term changes in neural circuits that underlie enduring modifications in brain structure and function^1,2^. Environmental enrichment (EE) strongly accelerates the maturation of sensory systems^3–10^, acting on molecular factors involved in cerebral cortex development and plasticity such as insulin-like growth factor 1 (IGF-1), brain-derived neurotrophic factor (BDNF) and GABAergic transmission^3,4,11–16^. Interestingly, an enriched experience restricted to the pre-weaning period is already sufficient to influence brain development^4,17^ and contributes to shape inter-individual differences in stress vulnerability and anxiety-like behavior with IGF-1 standing out as a crucial early EE mediator^4,17,18^.

On the opposite side of a continuum for the quality of environmental stimuli, empirical research produced overwhelming evidence that early environmental impoverishment, in terms of both institutional rearing and disadvantageous living conditions, could have adverse influence on the development of children^19,20^. Despite the number of experiments showing the effect of single sensory deprivation on the development of central processing^21,22^, direct evidence that impoverished living conditions can affect the functional and structural development of the nervous system is still lacking since most studies on the effects of impoverished environment (IE) were focused on adult animals. These studies showed that IE is able to modify animals’ behavior leading to impairments of cognitive functions^23–27^ paralleled by anatomical^1,24,28–33^ and molecular changes in the brain^1,26,34^. The influence of IE on the maturation of the nervous system has been described only in the form of isolate-rearing effects, with studies showing that post-weaning deprivation of social interactions in rodents and non-humane primates permanently affects brain neuronal morphology^35–38^, hippocampal functions^39–41^ and behavior^42–46^. Nothing is known on the effects of pre-weaning IE taking the form of an overall reduction in the richness of sensory-motor stimulation.

The aim of this study was to shed light on the impact of early sensory-motor impoverishment on developmental trajectories of the central nervous system and to start investigating possible molecular factors affected by IE. We developed a protocol of IE in which animals live from birth, with the mother and the other pups, in a condition of impoverished sensory stimulation and reduced motor activity. Thus, our investigation is not concerned with the effects of juvenile social deprivation, but it is targeted to understand how early impoverishment of rearing environment, including the pre-weaning period, might reverberate on the developmental trajectories of neuronal networks. We used the visual development as a model of brain maturation not only because it is a paradigmatic example of experience-dependent development and is profoundly affected by early EE, but also because it is increasingly evident that impoverished developmental conditions such as those of institutionalized children can lead to delays and deficit in sensory development^47^.

We focused on IGF-1 since it has been recognized as one of the critical factors shaping early neural development *in vivo*, with transient enhancements of IGF-1 expression within different time windows in distinct brain regions^48,49^. Moreover, we have recently shown that IGF-1 levels are increased early in the postnatal visual cortex of EE rats and this is a crucial event for setting in motion the developmental program induced by EE^12,13,50^. IGF-1 seems to be upstream of BDNF and inhibitory GABAergic circuit development^12,13,50^ and mediates not only EE effects on visual development but also the effects of an early enriched experience provided by massage, which recapitulates key features of maternal care in rat pups and in human babies^17^.

We found that early impoverishment strongly delays cortical development with detrimental consequences at the level of sensory, motor and cognitive functions. In the visual cortex of IE pups, the functional changes were accompanied by a significant reduction of IGF-1 protein associated with a decreased GAD67 expression and delayed myelination of nerve fibers.

## Results

### Developmental body-weight gain is delayed by impoverished environment

**Figure 1:**
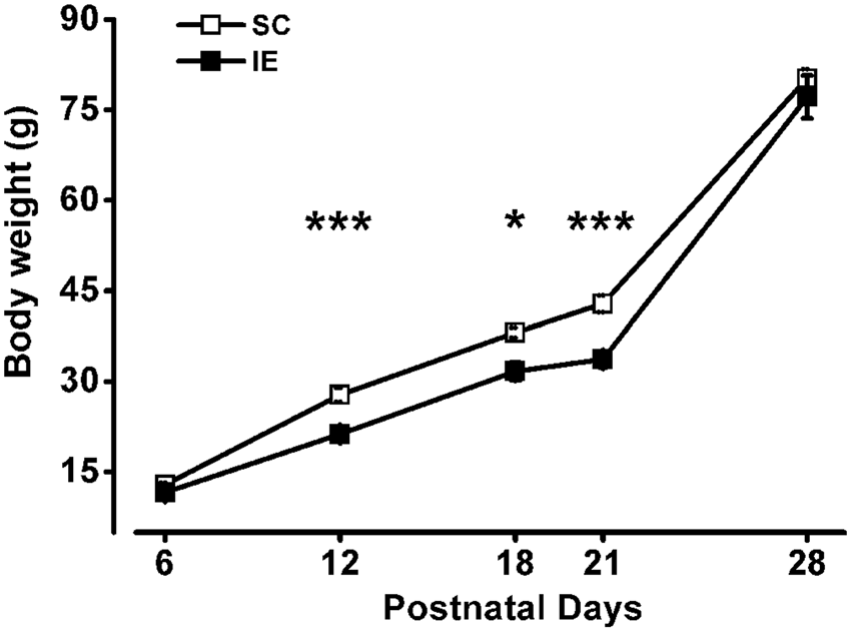
Developmental body-weight gain is delayed by impoverished environment. Body weight of IE animals was significantly decreased compared to that of SC rats at P12 (SC P12, n = 27; IE P12, n = 32; Two Way ANOVA, post-hoc Holm-Sidak method, p < 0.001), P18 (SC P18, n = 10; IE P18, n = 16; p < 0.05) and P21 (SC P21, n = 49; IE P21, n = 37; p < 0.001); no significant difference was found in the weight of SC and IE animals at P6 (SC P6, n = 50; IE P6, n = 36; Two Way ANOVA, post-hoc Holm-Sidak method, p = 0.35) and P28 (SC P28, n = 20; IE P28, n = 15; post-hoc Holm-Sidak method, p = 0.15). Symbols represent average values ± SEM. Error bars are smaller than the size of the symbols for most data points. Asterisks denote significant difference (*p < 0.05; ***p < 0.001).

The quality of the environment can affect fetal development^50–54^ and maternal care has a significant effect on the rate of weight gain^50,55^. We measured body weight of standard (SC) and IE animals at different ages during pre- and post-weaning life (P6, P12, P18, P21 and P28). While no difference was detected in the weight of animals early after birth (P6), IE rats displayed a significantly lower body weight in the period between P12 and P21. Eventually, the weight of IE animals became not different from that of SC animals at P28 (Fig. 1). These results suggest that impoverished environment influences somatic pup development during the pre-weaning period.

### Delayed functional maturation of visual cortex in IE rats

**Figure 2:**
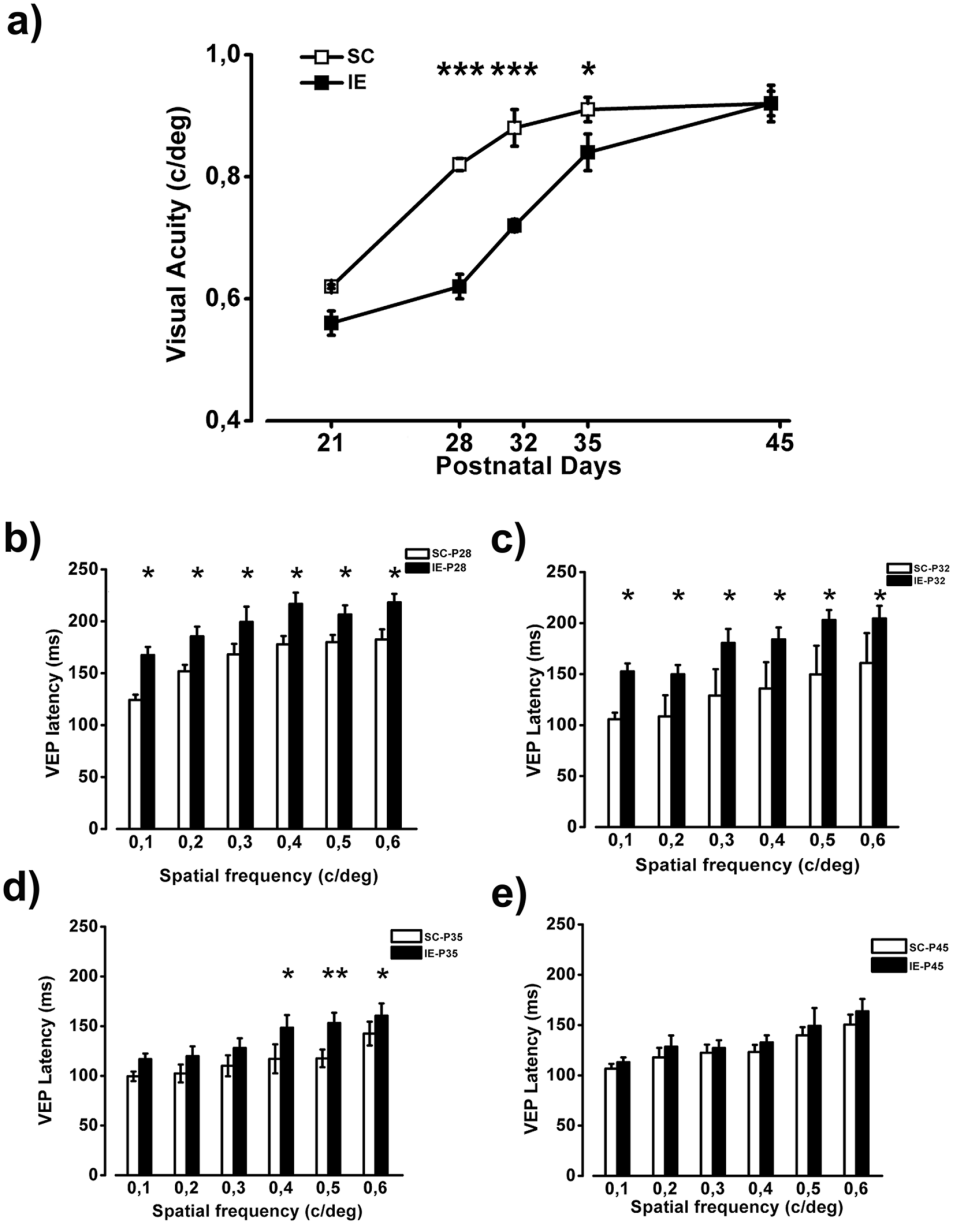
Delayed functional maturation of visual cortex in IE rats. (**a**) Visual acuity (VA) of IE animals was significantly decreased compared to that of SC rats at P28 (SC P28, n = 6; IE P28, n = 6; Two Way ANOVA, post-hoc Holm-Sidak method, p < 0.001), P32 (SC P32, n = 5; IE P32, n = 7; p < 0.001) and P35 (SC P35, n = 5; IE P35, n = 6, p < 0.05) but not at P21 (SC P21, n = 3; IE P21, n = 6; Two Way ANOVA, post-hoc Holm-Sidak method, p = 0.09) and P45 (SC P45, n = 6; IE P45, n = 5; post-hoc Holm-Sidak method, p = 0.81). Symbols represent average VA values ± SEM. Error bars are smaller than the size of the symbols for some data points. (**b**,**c)** At P28 and P32 VEP latency of IE rats was increased with respect to SC animals at 0.1 c\deg, 0.2 c\deg, 0.3 c\deg, 0.4 c\deg, 0.5 c\deg and 0.6 c\deg (Two way RM ANOVA, post-hoc Holm-Sidak method, p < 0.05 for all comparisons). (**d**) At P35 P100 latency of IE rats was increased with respect to SC animals at 0.4 c\deg, 0.5 c\deg and 0.6 c\deg (Two way RM ANOVA, post-hoc Holm-Sidak method, p < 0.05 for all comparisons, except for p < 0.01 at 0.5 c/deg). **e)** VEP latencies at P45 were not different between SC and IE animals (Two way RM ANOVA, p = 0.42). Histograms represent latency average values ± SEM. *p < 0.05; **p < 0.01; ***p < 0.001.

Visual acuity is a robust index of maturation of the visual cortex and its development proceeds in parallel with the maturation of visual circuits, being completed, in rats, around at P45^4,8^. We measured visual acuity development in SC and IE rats using electrophysiological recordings of VEPs. As shown in Fig. 2a, the maturation of visual acuity in IE animals was strongly delayed with respect to SC rats, with significantly lower values at P28, P32 and P35. In particular, at P28 visual acuity of IE rats was lower than that of SC animals by 25%. While final visual acuity values did not differ between SC and IE animals, mature values were achieved 10 days later in IE than in SC rats.

The latency of the first VEP component (P/N1) is an important indicator of the early development of visual cortex, tightly associated with the formation and myelination of neural circuits^56–58^. We measured at different ages VEP latency for spatial frequencies (SFs) in the range 0.1–0.6 c/deg, that is from the low to the high SF zone of the contrast sensitivity curve. Impoverished animals showed a significant delay in the maturation of VEP latencies: higher VEP latencies with respect to SC animals were observed for all SFs tested, at both P28 and P31 (Fig. 2b,c); at P35, IE animals still showed higher latencies for high SFs, while they displayed normal latencies for low SFs (Fig. 2d). At P45, VEP latencies became not significantly different between IE and SC rats (Fig. 2e).

These results demonstrate that cortical development is sensitive to the reduction of stimulation experienced in an impoverished environment.

### Impairment of motor functions and memory retention in IE rats

**Figure 3:**
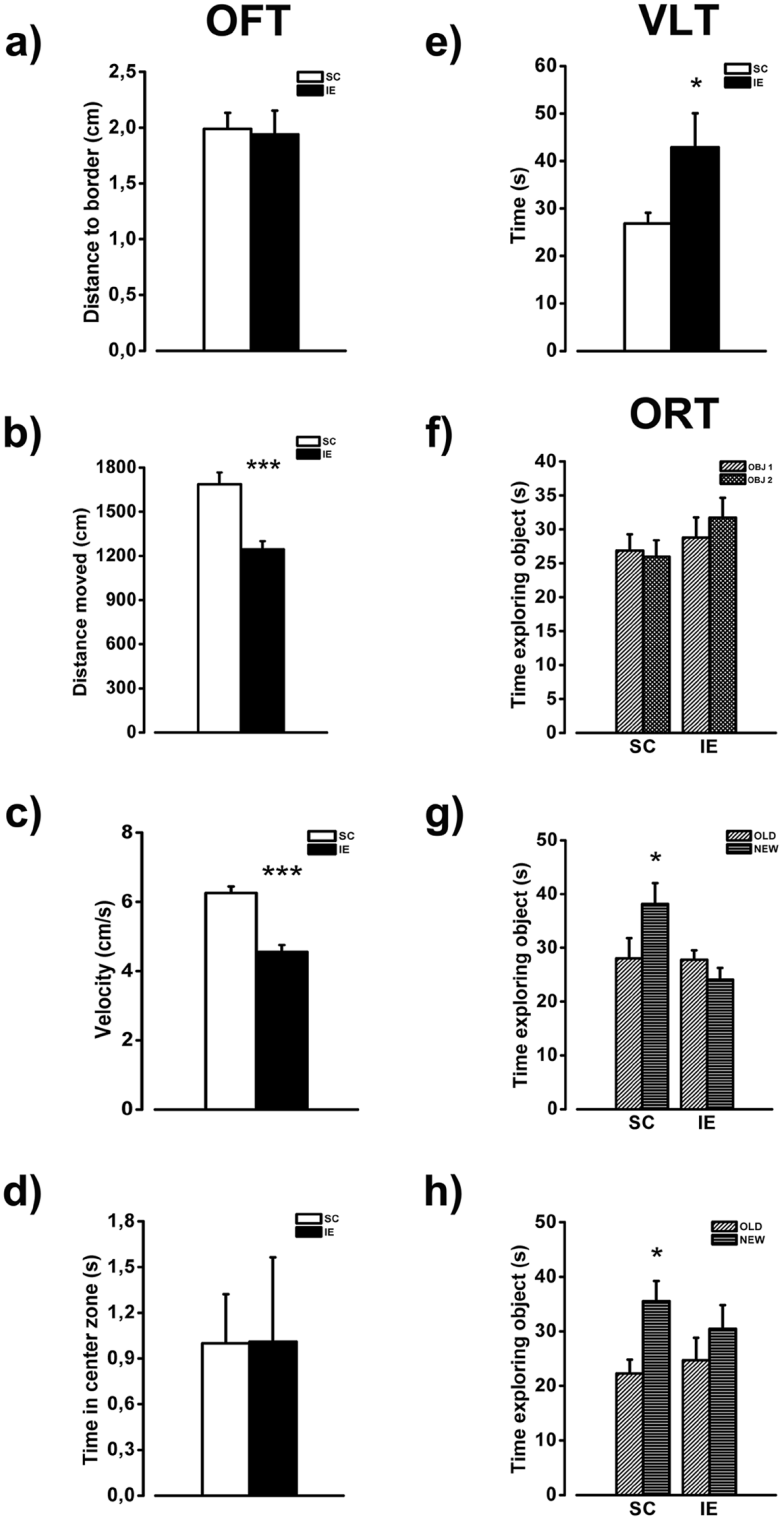
Behavioral assessment of motor and cognitive functions in IE rats. (**a–d**) Open field test (OFT). (**a**) IE and SC animals performed similarly in the open field arena when the exploration was measured as mean distance to the arena border (SC P30, n = 16; IE P30, n = 16; t-test, p = 0.84). (**b**,**c**) T-test revealed that the IE behavior was different from that of SC animals both in terms of total distance moved and mean velocity in the open field maze (t-test, p < 0.001 and p < 0.001 respectively). (**d**) The percentage of time spent in the center part of the arena was not different between SC and IE rats (t-test, p = 0.933). (**e**) Assessment of sensorimotor coordination through vertical ladder climbing (VLT) confirmed a motor deficit in IE animals respect to SC (SC P30, n = 12; IE P30, n = 12; t-test, p < 0.05). (**f–h**) Object recognition task (ORT). (**f**) Both SC and IE rats spent equal amount of time exploring the two objects (OBJ1 and OBJ2) during the familiarization phase of the Object Recognition Test (SC P30, n = 16; IE P30, n = 16; paired t-test, p = 0.46 and p = 0.097). (**g**,**h**) SC showed a good information retention after both 1 and 24 hours (paired t-test, p < 0.01 and p < 0.001 respectively), while IE animals did not recognize the new object (NEW) with respect to the old one (OLD) either after 1 or after 24 hours (paired t-test, p = 0.06 and p = 0.08, respectively). Histograms represent average values ± SEM. *p < 0.05; **p < 0.01; ***p < 0.001.

To assess whether the exposure to an impoverished environment results in detrimental effects on general motor activity and anxiety-related behavior, we tested P30 IE rats in the open field arena^59^. While we did not find any difference in anxiety levels (Fig. 3a,d), IE subjects turned out to exhibit reduced motor activity: (i) total distance moved in the open field and (ii) mean velocity, indeed, were significantly reduced in the IE group (Fig. 3b,c). To better analyze sensorimotor coordination, we also evaluated IE rats in the vertical ladder climbing^60^. This motor-specific test showed that IE rats require significantly more time to reach the top surface of the ladder in comparison to SC animals, confirming the general impairment of motor abilities (Fig. 3e).

Then, we assessed declarative memory in the object recognition task (ORT), a test based on the spontaneous tendency of rodents to spend more time exploring a novel object than a familiar one^61,62^. While no difference was present between IE and SC rats in the familiarization phase, IE P30 animals showed impaired memory retention at both 1 and 24 hours in comparison with age-matched SC rats, suggesting that impoverished rearing conditions hamper memory function development (Fig. 3f–h).

### IE affects IGF-1 expression in the viNsual cortex

**Figure 4:**
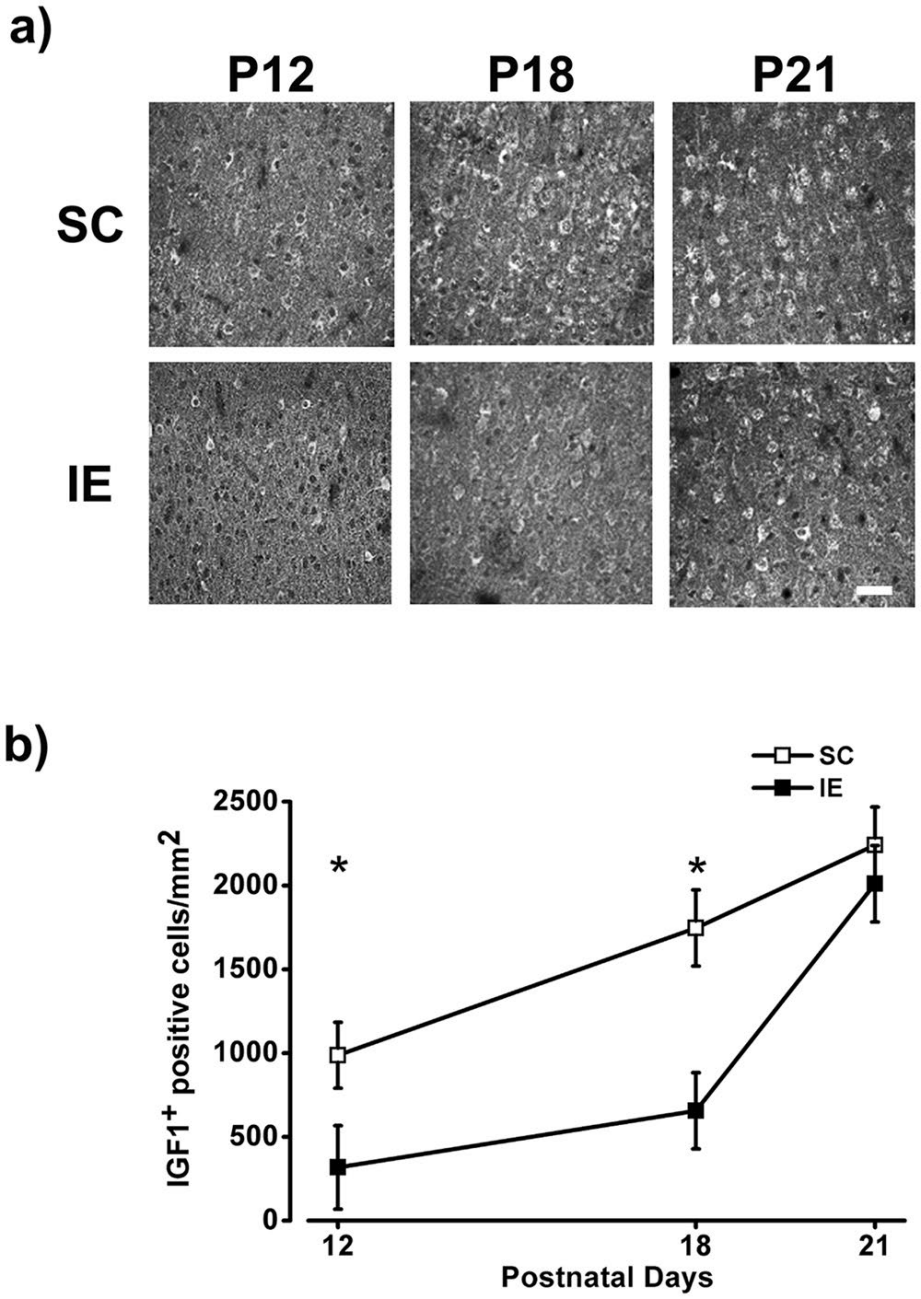
IE affects IGF-1 expression in the visual cortex. (**a**) Representative examples of IGF-1 labeling from fields taken in the layers V/VI of the visual cortex of SC and IE rats at P12, P18 and P21. Calibration bar: 50 µm. (**b**) Number of IGF-1 positive (IGF-1^+^) cells/mm^2^ in the visual cortex of SC and IE animals. IGF-1^+^ cell density was lower in IE animals at P12 (SC P12, n = 7; IE P12, n = 4) and P18 (SC P18, n = 6; IE P18, n = 6; Two Way ANOVA, post-hoc Holm-Sidak method, p < 0.05 for both comparisons). The number IGF-1^+^ cells did not differ between SC and IE at P21 (SC P21, n = 5; IE P21, n = 5; Two Way ANOVA, post-hoc Holm-Sidak method, p = 0.48). Symbols represent average values ± SEM. *p < 0.05.

To shed light on the molecular mechanisms underlying the influence of IE on cortical development, we focused on IGF-1, which has been implicated in prenatal and postnatal events of brain maturation^63,64^ and it is known to be one critical mediator of the effects of environmental stimulation on brain development and plasticity^11,13,50,65–68^. IGF-1 protein was revealed by means of an immunohistochemical protocol repeatedly used to analyze IGF-1 presence in the central nervous system^69,70^, and in particular to characterize its developmental trajectory in the visual cortex^11,13^. The number of IGF-1 positive cells per mm^2^ was significantly lower in IE than in SC rats at P12 and P18, while no difference in IGF-1 levels was present between SC and IE rats at P21(Fig. 4). Importantly, the reduction of IGF1-positive cells at P12 (Two way RM ANOVA effect of housing condition p < 0.001, post hoc Holm Sidak method p < 0.001 for all layers) and P18 (Two way RM ANOVA effect of housing condition p < 0.001, post hoc Holm Sidak method p < 0.01 for layers I-II and III-IV, p < 0.001 for layer V-VI) was evident in all cortical layers. The decrease in IGF-1 positive cells caused by IE was not due to a decrease of neuronal density in the visual cortex of IE animals: indeed, the number of NeuN positive cells did not differ between the two experimental groups (Fig. S1). These results clearly indicate that IE affects the developmental time course of IGF-1 protein levels in the visual cortex, causing a delay of IGF-1 expression with respect to animals housed in standard condition.

### IE affects the density of inhibitory neurons in the visual cortex

Maturation of intracortical inhibitory circuits has a major role in visual acuity development. The developmental increase of visual acuity, indeed, is correlated with a progressive reduction of visual cortical receptive field size, which in turn is strictly dependent from intracortical inhibition levels^71–73^. Moreover, it has been reported that IGF-1 regulates the development of GABAergic intracortical inhibition^11,13^ and affects the production and migration of GABAergic neurons^74^.

**Figure 5:**
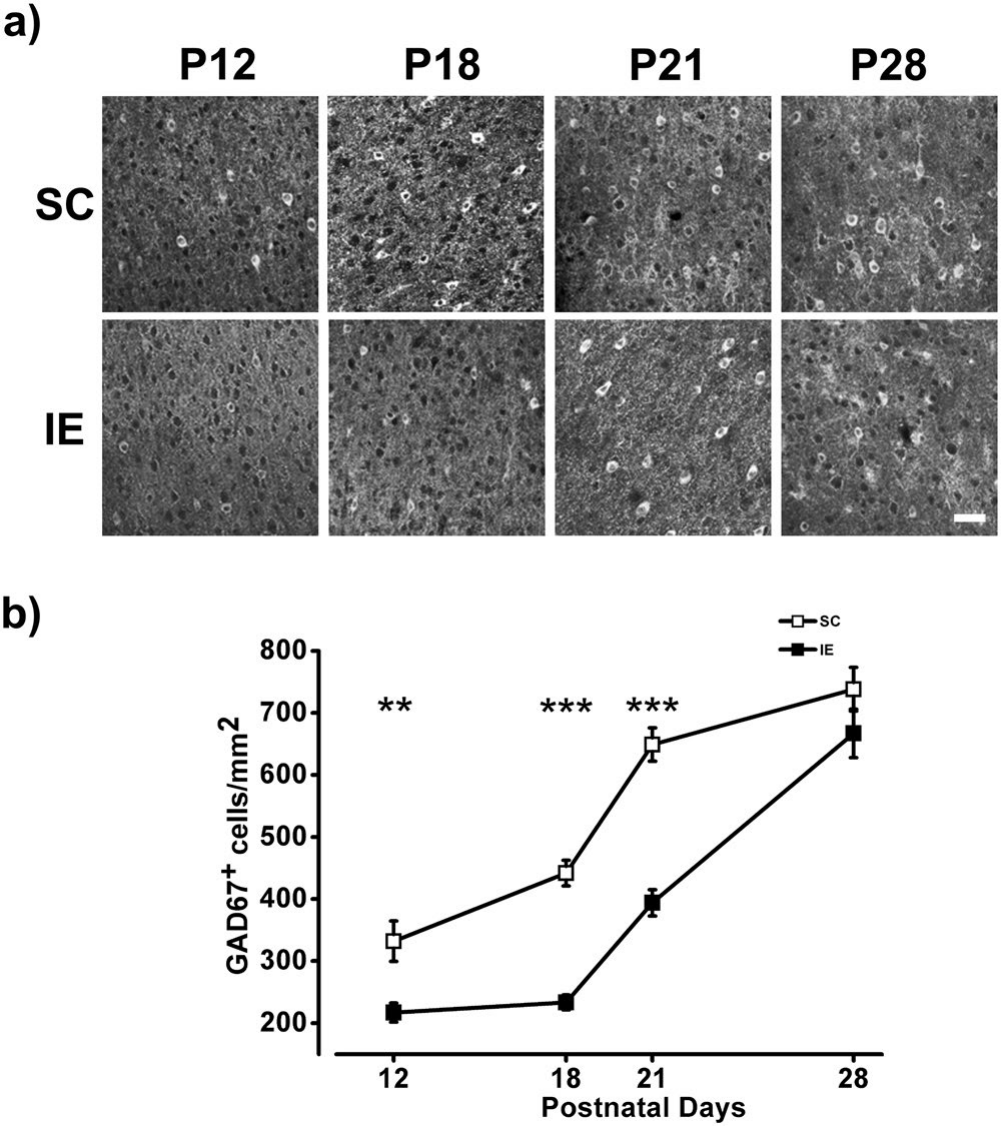
IE affects the density of inhibitory neurons in the visual cortex (**a**) Representative fields of GAD67 labeling taken in the layers V/VI of the visual cortex of SC and IE rats at P12, P18, P21 and P28. Calibration bar: 50 µm. (**b**) Quantification of GAD67 levels, in terms of number of GAD67 positive (GAD67^+^ cells/mm^2^), in the visual cortex of SC and IE animals. GAD67^+^ cell density was lower in IE animals at P12 (SC P12, n = 4; IE P12, n = 4; Two Way ANOVA, post-hoc Holm-Sidak method, p < 0.01), P18 (SC P18, n = 6; IE P18, n = 6; p < 0.001) and P21 (SC P21, n = 3; IE P21, n = 3; p < 0.001). The number GAD67 positive cells did not differ between SC and IE at P28 (SC P28, n = 3; IE P28, n = 3; Two Way ANOVA, post-hoc Holm-Sidak method, p = 0.1). Symbols represent average values ± SEM. **p < 0.01; ***p < 0.001.

We hypothesized that the slowdown effect in visual acuity development observed in IE animals could be due to a delayed maturation of intracortical inhibitory circuits. Thus, we investigated the developmental expression of GAD67 protein levels in the visual cortex of SC and IE rats from P12 to P28. The density of GAD67 positive cells increased between P12 and P28 in both SC and IE rats, but with a significant delay for the latter group: GAD67 levels were significantly lower in IE animals with respect to SC group at P12, P18 and P21 (Fig. 5). Also in this case the reduction in the number of GAD67-positive interneurons was significant in all cortical layers (P12: Two way RM ANOVA effect of housing condition p < 0.01, post hoc Holm Sidak method p < 0.05 for layers I-II and III-IV, p < 0.01 for layer V-VI; P18: Two way RM ANOVA effect of housing condition p < 0.001, post hoc Holm Sidak method p < 0.001 for layers I-II and III-IV, p < 0.01 for layer V-VI; P21: Two way RM ANOVA effect of housing condition p < 0.001, post hoc Holm Sidak method p < 0.001 for layers I-II and V-VI, p < 0.001 for layer III-IV). At P28 the number of GAD67 positive neurons was not significantly different between SC and IE rats (Fig. 5).

### IE delays axonal myelination in the visual cortex

Since myelination is closely associated with axon maturation and is essential for establishing an efficient neuronal signaling network^75–77^, we investigated the developmental pattern of myelin basic protein (MBP) staining in the visual cortex of SC and IE rats.

**Figure 6:**
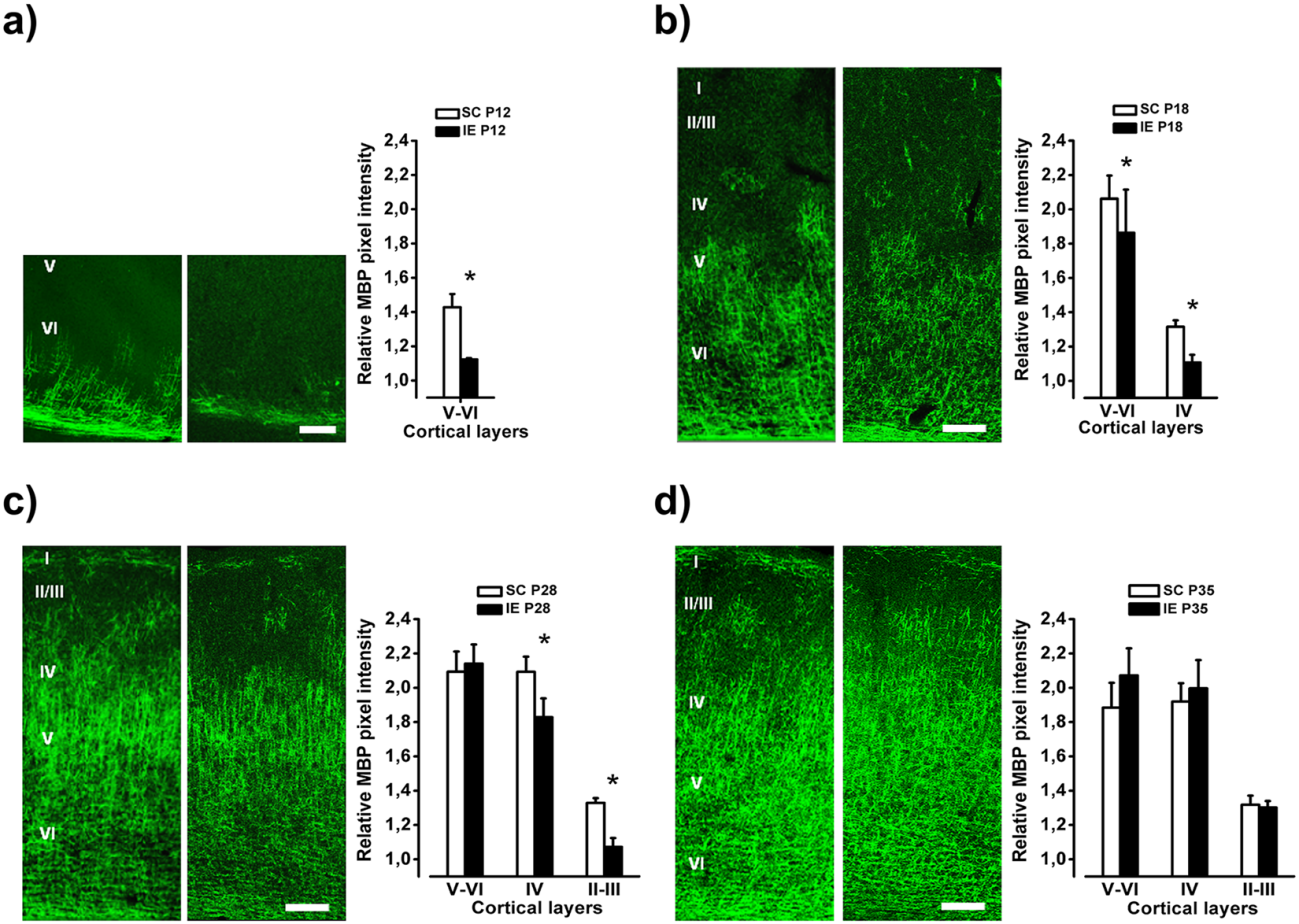
IE delays axonal myelination in the visual cortex. (**a**) Left: Example of MBP labeling from fields taken in layers V/VI of the visual cortex of P12 SC and IE rats. Calibration bar: 50 μm. Right: Quantitative analysis of MBP immunofluorescence intensity in layers V/VI of the visual cortex of P12 animals. SC animals showed higher MBP expression in comparison to IE animals in the layers V/VI (SC P12, n = 4; IE P12, n = 6; Mann-Whitney Rank Sum Test, p < 0.05). (**b**–**d**) Left: Example of MBP labeling from fields taken through all layers of the visual cortex in P18, P28 and P35 SC and IE rats. Calibration bar: 100 μm. Right: Quantitative analysis of MBP immunofluorescence intensity in all layers of the visual cortex of P18, P28 and P35 animals. At P18 SC animals showed higher MBP immunofluorescence in layers V/VI and IV (SC P18, n = 5; IE P18, n = 6; Two way RM ANOVA, Post-hoc Holm-Sidak method, p < 0.05). At P28 SC animals showed higher MBP expression in comparison to IE animals in the layers IV and II-III (SC P28, n = 6; IE P28, n = 6; Two way RM ANOVA, Post-hoc Holm-Sidak method, p < 0.05). The layers V-VI were completely myelinated and MBP expression did not differ between groups (p = 0.74). At P35 MBP immunofluorescence did not differ between SC and IE animals in any cortical layer (SC P35, n = 5; IE P35, n = 6; Two way RM ANOVA, p = 0.63). Histograms represent average values ± SEM. *p < 0.05.

During cerebral cortex development, myelination starts from the deepest layers and proceeds towards the superficial ones^78^. We observed that layer-specific levels of intracortical myelin matured at a slower pace in IE than in SC animals. At P12, layer VI was totally myelinated in SC animals, while only partially in IE rats. One week later (P18), the relative amount of myelin was still lower in IE visual cortex with respect to SC animals both in layer IV and in layers V-VI (Fig. 6). At P28, layers V-VI were completely myelinated in both groups, but the magnitude of myelination was significantly greater in SC rats in layers II-III and in layer IV. Eventually, the intensity of immunostaining for MBP did not differ between IE and SC animals at P35 (Fig. 6). Thus, IE causes a delay in cortical myelin maturation.

### Hypophosphorylation of rpS6 in IE brain

mTOR/PI3K- and ERK-dependent protein synthesis are crucial for neuronal development and expression of synaptic plasticity^79–81^. Moreover, it has been shown that activation of IGF-1 receptor can initiate a molecular cascade resulting in the stimulation of PI3K-AKT-mTOR and MAP kinase pathway^82,83^. To investigate whether defects of these intracellular pathways might occur in the brain of IE rats, we examined the immunolocalization of phosphorylated (p) ribosomal protein (rp) S6, a converging target of both ERK and mTOR/PI3K activity upon protein synthesis^84^. Sections were probed with two specific antibodies: the first recognizes rpS6 only when activated at Ser235/236, while the second recognizes the activated form of rpS6 at Ser240/244 sites.

**Figure 7:**
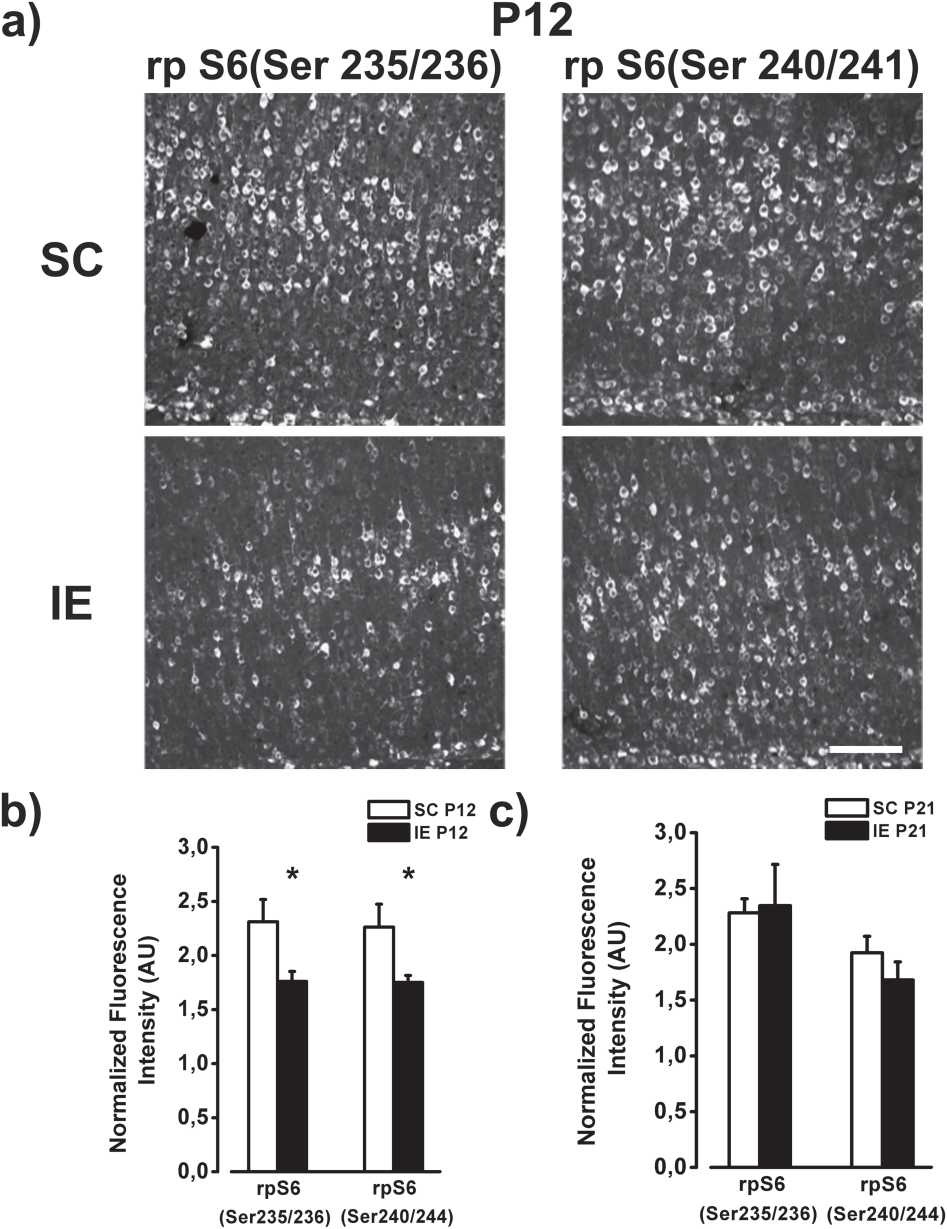
Hypophosphorylation of rpS6 in IE brain. (**a**) Example of rp S6 labeling (Ser235/236 and Ser 240/241) from fields taken in the layers V/VI of the visual cortex of P12 SC and IE rats. Calibration bar: 100 µm. (**b**) Quantitative analysis of rp S6 immunofluorescence intensity in the visual cortex of P12 animals. SC animals showed higher rpS6 expression in comparison to IE animals both for the site Ser235/236 and for the site Ser240/244 (SC P12, n = 5; IE P12, n = 5, Two way ANOVA, post-hoc Holm-Sidak method, p < 0.05). (**c**) At P21 rp S6 expression did not differ between SC and IE animals (SC P21, n = 5; IE P21, n = 6; p = 0.48). Histograms represent average values ± SEM. *p < 0.05.

We analyzed two ages, P12, when IGF-1 is lower in IE with respect to SC rats, and P21, when no difference between IE and SC animals is found. At P12, we found a robust decrease in p-rpS6 immunoreactivity throughout the visual cortex of IE rats compared with SC animals (Fig. 7). For both biomarkers the reduction of immunoreactivity was evident across all cortical layers (p-rpS6 Ser 235/236:Two way RM ANOVA effect of housing condition p < 0.01, post hoc Holm Sidak method p < 0.05 for layers I-II and V-VI, p < 0.01 for layers III-IV; p-rpS6 Ser 240/244:Two way RM ANOVA effect of housing condition p < 0.05, post hoc Holm Sidak method p < 0.05 for all layers). These results indicate that rpS6 function is impaired in IE rats. Conversely, the intensity of p-rpS6 immunostaining did not differ between IE and SC animals at P21, suggesting that IE affects the activation of rpS6 with a time course similar to that reported for IGF-1.

### Influence of mother-offspring interaction in the effects of IE on brain development

Maternal care is one of the most important early sources of experience for the developing offspring, regulating physical growth and promoting neural maturation of brain structures from rodents to primates^2,85^.

To investigate whether the developmental delay observed in IE pups might be dependent from lower levels of maternal care provided to impoverished pups, we performed a detailed analysis of maternal behavior in IE and SC dams. Behavioral observations showed no attenuation in maternal care levels received by IE pups during the first ten days of postnatal life with respect to SC animals, with IE dams even showing an increase in the percentage of time spent in the nest and in passive nursing (Fig. S2). Thus, the effects of early IE on visual cortex development cannot be attributed to lower maternal care in IE conditions.

**Figure 8:**
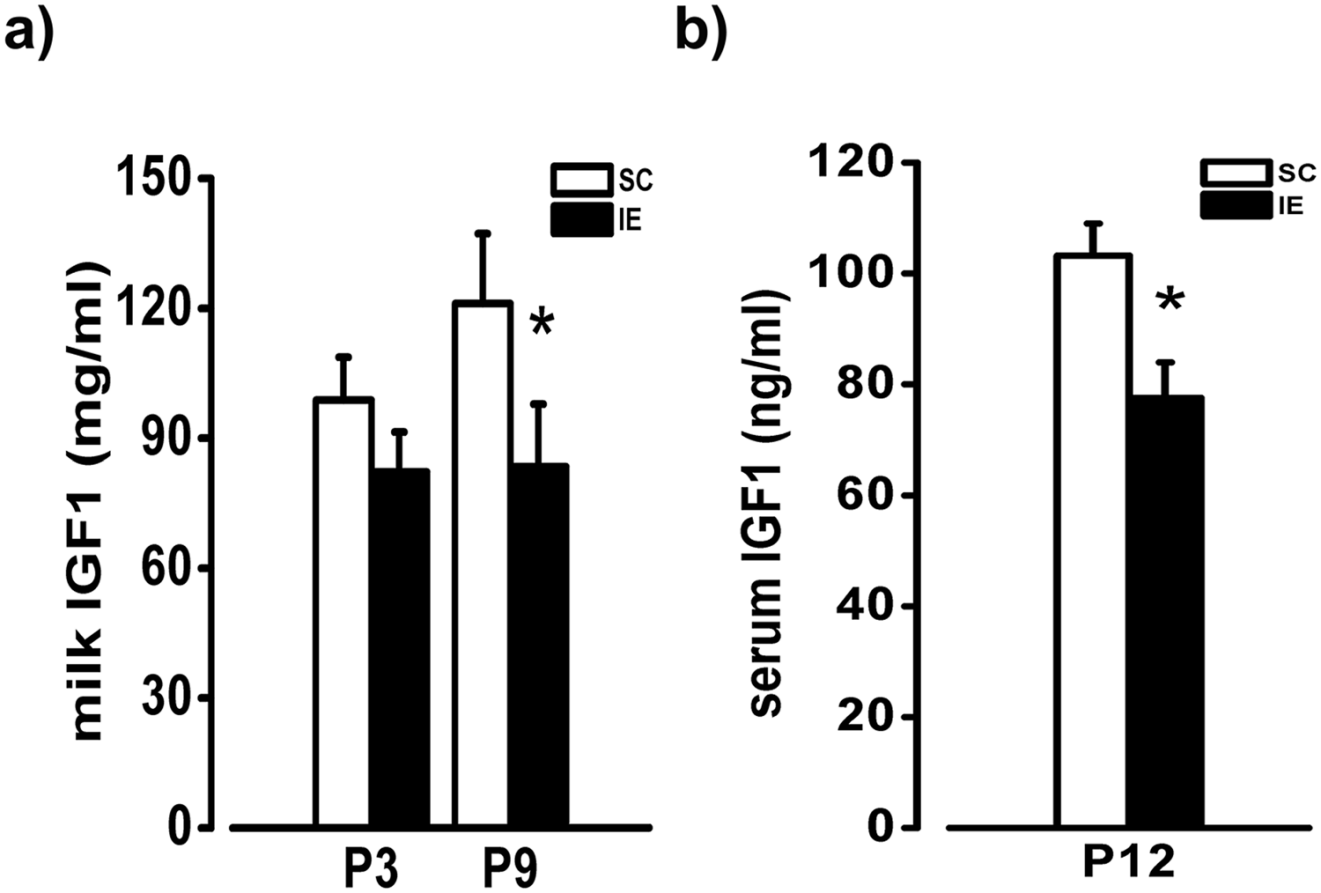
Decreased IGF-I concentration in the maternal milk and in pup serum. (**a**) RIA determination of IGF-I concentration in the milk of SC and IE dams. T-test revealed a difference at P9 (SC, n = 8; IE, n = 7; p < 0.05), but not at P3 (p  =  0.105) between SC and IE animals. (**b**) IGF-I levels in the serum of SC and IE pups at P12. T-test revealed a significant difference between SC and IE animals (SC, n = 9; IE, n = 9; p < 0.05). Histograms represent average values ± SEM. *p < 0.05.

An alternative source for the observed alteration of developmental trajectories in IE pups could be the presence of lower amounts of nutrients and growth factors in the maternal milk under impoverished conditions. Indeed, the early beneficial effects of enriched experience are known to be mediated by IGF-1^13,18^, with higher levels of IGF-1 in enriched maternal milk promoting IGF-1 production in the brain of enriched pups^50,53^. Thus, we measured IGF-1 levels in the milk dams 3 and 9 days after delivery, and in the serum of pups at P12. We found lower levels of IGF-1 in the milk of IE compared to SC mothers at the ninth day postpartum (Fig. 8a), suggesting that IE actually causes a decreased transfer of nutrients to developing pups. Accordingly, the amount of IGF-1 detected in the serum of IE pups was significantly decreased with respect to SC animals (Fig. 8b).

## Discussion

The aim of our study was to shed light on the impact of early sensory-motor impoverishment, in absence of social deprivation of the pups, on developmental trajectories of the central nervous system and to start investigating possible molecular factors affected by IE. Our investigation was not concerned with the effects of juvenile social deprivation, but was targeted to understand how early sensory-motor impoverishment of rearing environment, including the pre-weaning period, might affect the developmental trajectories of neuronal networks. Indeed, a direct demonstration that environmental impoverishment can affect the developmental trajectories of the nervous system is still missing.

We demonstrated that rearing rats from birth in an impoverished environment characterized by a reduction of sensorimotor stimulation leads to a marked delay of functional properties of visual system development, including visual acuity and the latency of visual cortical responses to the sensory input. These functional changes were accompanied by alterations in the window of developmental expression of several molecular factors known as general drivers of functional maturation of neural circuits, such as IGF-1, GAD67 and MBP.

These data allow a detailed understanding of how early environmental impoverishment, and not only social deprivation, affects the maturation of neuronal networks. Even if we mainly focused on the visual cortex, it is very likely that the effects provided by the impoverishment protocol are not restricted to the developing visual system, as suggested by the impairment of cognitive and motor functions in young animals. Similarly, enriched experience has been reported to have beneficial effects on cortical circuits beyond the visual system^3,17,50^. Even if all changes observed are transient, the delaying effect of IE is conspicuous. Noteworthy, the delay in visual acuity development is by 10 days, which is a highly significant lapse of time given that the entire process of visual acuity maturation in rats lasts only 30 days.

There was a good correlation between the functional and cellular/molecular developmental delay caused by IE. The maturation of visual acuity is strictly correlated with a decrease of receptive field size of neurons in the primary visual cortex^71^ and the development of intracortical inhibitory circuitry^72,73,86^. Consistently, the cortex of IE rats displayed a slower maturation of GAD67-expressing interneurons that could impinge on the formation and refinement of visual cortical receptive fields. The latency of VEPs, another very reliable index of the developmental stage of the brain both in animal models and in humans^17,87^, clearly depends from the conduction velocity in visual pathways. The slower decrease in latency of VEP responses in IE animals correlated well with the slower myelination rate in different layers of IE visual cortex and could be predictive of an alteration in cortical circuitries’ formation and synaptic transmission maturation.

Some of the protein changes in the brain of IE rats were observed at a very early age, when rodent life is characterized by the absence of a direct interaction between the pup and the external environment and the mother is the most important source of sensory experience^88,89^. In particular, IGF-1, BDNF, GAD and visual acuity development correlate with the level of maternal care, with pups receiving lower level of care showing delayed visual acuity development and lower IGF-1, BDNF and GAD levels with respect to enriched pups, who receive higher amounts of care^3,11,50,90^. If IE were to affect maternal behavior, the ensueing reduced maternal care would likely delay visual acuity development and reduce observed protein levels. However, direct behavioral observations of maternal care under IE ruled out that impoverished living conditions might have affected maternal behavior leading to reduced maternal care. Since increased maternal stress leads to reduction in maternal care, this result indirectly suggests that IE did not affect stress levels in dams^50,91^.

The absence of reduced maternal care in IE also indicates that the employed IE paradigm does not model early social deprivation. Accordingly, IE caused a delay in the developmental time course of brain function and underlying molecular factors but not a change in their final level, whereas the lack of proper maternal care is known to lead to long-term maladaptive effects in the cognitive and emotional domain^92^.

It is well-known that IGF-1 plays a critical role in shaping neural development *in vivo* with transient enhancements of its expression in distinct brain regions^49,93^, and that the modulation of IGF-1 levels is crucially involved in mediating the effects of environmental enriched experience on postnatal retinal and cortical maturation^4,11,13,17,18^. We showed that IGF-1 protein levels are significantly decreased at early postnatal ages in the visual cortex of animals reared in impoverished conditions. We hypothesize that this event could underlie the detrimental effects of IE on visual cortex maturation. IGF-1 signaling, indeed, plays trophic functions in neural development, affecting proliferation, survival and differentiation of brain cells, synapse maturation and circuit formation^49,93,94^. We propose that reduced IGF-1 in the pup brain is caused by reduced IGF-1 in the milk of IE mothers. It is known that the brain receives a conspicuous IGF-1 input from the periphery^49,69^ and a decreased transfer of nutrients from the mother to the pups could in turn lead to lower amounts of IGF-1 autonomously produced by the pups^7,13,50,95^. Consistently, we detected significantly lower levels of IGF-1 in the serum of IE pups. This result is also in line with the lack of difference in IGF1 mRNA expression reported for the visual cortex of EE and SC animals^13^.

The effects of IGF-1 on visual cortical development are likely to be primarily related to the maturation of GABAergic system. It is likely that the reduction of GAD67 expression observed between P12 and P21 in IE rats is causally dependent on reduced IGF-1 levels in the IE visual cortex. A direct action of IGF-1 on inhibitory interneurons is possible thanks to the documented presence of IGF-1 receptors in GABAergic neurons^11^; thus IE rearing, acting through reduced IGF-1, may cause a slower maturation of cortical visual acuity by delaying the development of inhibitory GABAergic transmission. The delaying action of IE is not restricted to maturational processes occurring at the neuronal cell level. We showed that IE strongly delays axonal myelination in the visual cortex, confirming that early negative experience can result in white matter alterations and defective oligodendrocyte maturation^96,97^. IGF-1 is a potent agent in promoting the growth, differentiation and survival of oligodendrocyte lineage cells and in stimulating myelination during development and following injury^98–103^. A direct interaction of IGF-1 and oligodendrocytes has been demonstrated by the reduction of oligodendrocyte precursors and mature oligodendrocytes in mutant mice with conditional deletion of IGF-1 receptor in the cells of oligodendrocyte lineage^104^. Thus, we hypothesize that transient dysregulation of IGF-1 signaling in IE brain has detrimental effects on myelination, leading to a transient decrease of neuronal communication speed.

Finally, there are other molecules, whose expression is regulated by IE experience, that might play an indirect role in the context of brain plasticity, such as metabolic enzymes and proteins implicated in energy metabolism^34^. IGF-1 is an important regulator of general metabolism^105^ coordinating brain responses with respect to metabolic status^106^. The effects of IGF-1 on cell growth and metabolism during neural development are mostly mediated by the PI3K-AKT-mTOR and by the MAP kinase pathway, resulting in the stimulation of protein synthesis via activation of rpS6^82,84^. Accordingly, we observed that the phosphorylation of rpS6 was reduced in the brain of IE animals and the dynamic of its reduction followed the delayed developmental trajectory of IGF-1 expression, suggesting that phospho-rpS6 levels are deregulated by the impairment of IGF-1 signaling.

Even if our data point to IGF-1 as a crucial player for the IE effects on brain maturation, we cannot rule out a contribution of other molecules, such as neurotrophins, different growth factors or immune system proteins, that have been linked to the regulation of brain developmental trajectories^107,108^.

Since the pioneering studies of René Spitz on the genesis of psychiatric disturbances in hospitalism^109^ it has been shown that early institutionalization has adverse influence on child development^19^. A large number of studies reported that children raised in orphanage, hospital or foundling home show marked delays in cognitive development, poorer physical growth, visual and hearing impairments^47^ and considerable deficits in social competence^19,110–112^.

One fundamental conclusion emerging from this work is that impoverished living conditions with limited sensory and motor stimulation, even if in association with normal social experience, cause a significant alteration of developmental trajectories leading to a prominent delay of brain maturation. In addition to the great relevance of social environment, these results demonstrate that an overall reduction in the richness of sensory-motor stimulation can also affect the functional and structural development of the nervous system, possibly inspiring a revision of the current guidelines for the welfare of institutionalized children. The lack of a permanent impairment in visual cortex maturation is possibly related to a late increase in the supply of molecular factors needed for neural circuit development. The reduced environmental stimulation provided by IE led to a substantial decrease of brain IGF-1 expression and we suggest that IGF-1 could be one of the main drivers in the molecular chain set in motion by IE, triggering the delay of intracortical inhibition maturation and, in parallel, the decrease of rpS6 phosphorylation and the defective myelination of visual cortical axons.

## Materials and Methods

### Animal housing

Long-Evans rats were maintained at 22 °C under a 12-h light–dark cycle (average illumination levels of 1.2 cd/m2). Food (4RF25 GLP Certificate, Mucedola) and water were available *ad libitum*. All experiments were approved by the Italian Ministry of Public Health and in line with guidelines for care and use of laboratory animals.

Females and males were equally distributed among experimental groups. To prevent litter-dependent effects, animals in each experimental group always came from different litters, with a minimum of three litters even for the smallest groups. Adult female rats were put with males (one male for every mating cage) in standard cages for reproduction (60 × 40 × 20 cm). Timed pregnant females were assigned to either impoverished (IE) or standard rearing conditions (SC). Parturition was checked one time a day, and the day of birth was considered postnatal day 0 (P0). Impoverished condition consisted of a small opaque cage (36.5 × 20 × 14 cm) with a filter covering the top and the long sides of the roof and white adhesive tape on the cage walls (Mod. 1284 L Eurostandard Type II, Tecniplast, Italy), leading to reduced maternal physical activity and reduced diversity of visual stimuli available to the animals. Every cage housed one single pregnant female and, after the delivery, one litter of pups with their impoverished dam. Standard housing consisted of a standard cage (40 × 30 × 20 cm), housing one litter of pups with their dam. In both experimental conditions, mothers were removed from the cages after weaning, when pups reached 21 days.

### *In Vivo* Electrophysiology

Visual evoked potentials (VEPs) were recorded from the binocular portion of the visual cortex (Oc1B). Rats were anesthetized with an i.p. injection of 20% urethane (Sigma; 0.7 ml/hg of body weight) and mounted in a stereotaxic apparatus. A portion of the skull (2 × 2 mm) overlying the OcB1 was drilled and the dura madre was removed. A resin-coated microelectrode (Harvard apparatus) with tip impedance of 2 MΩ filled with NaCl (3 M) was inserted perpendicularly to the stereotaxic plane into the Oc1B in correspondence of the vertical meridian representation and advanced 100 or 400 μm within the cortex. Typical visual stimuli were horizontal sinusoidal gratings of different spatial frequency generated by a VSG2/2 card (Cambridge Research System) and presented on a monitor suitably linearized by gamma correction. The display (25 cd/m^2^) was placed 20 cm in front of the animal and centered on the previously determined receptive fields. Electrical signals were amplified (10000 fold), band-pass filtered (0.1–100 Hz), digitized (12 bit resolution) and averaged (at least 50 events) in synchrony with the stimulus contrast reversal. Transient VEPs in response to abrupt contrast reversal (0.5 Hz) were evaluated in the time domain by measuring the peak-to-baseline amplitude and peak latency of the major component. Visual acuity was obtained by extrapolation to zero amplitude of the linear regression through the data points in a curve where VEP amplitude is plotted against log spatial frequency.

### Open field and object recognition test (ORT)

The apparatus consisted of a round arena (100 cm diameter, 40 cm height) constructed in poly(vinyl chloride) with white walls and floor. The rats received one session of 5-min duration in the empty arena to habituate them to the apparatus and test room. Animal position was continuously recorded by a video tracking system (Noldus Ethovision XT). The total movement and the velocity of the animal were automatically computed. Rat activity during this habituation session was analyzed for evaluating the behavior in the open field arena. The ORT consisted of two phases: sample and testing phase. During the sample phase, two identical objects were placed in diagonally opposite positions in the arena, approximately 12 cm from the walls, and rats were allowed 5 min to explore the objects, then they were returned to their cage. The objects to be discriminated were made of plastic, metal, or glass material and were too heavy to be displaced by the rat. The testing phase was performed either 1 h or 24 h after the sample phase. One of the two familiar objects was replaced with a new one, while the other object was replaced by an identical copy. The objects were placed in the same locations as the previous ones. The rats were allowed to explore objects for 5 min. To avoid possible preferences for one of two objects, the choice of the new and old object and the position of the new one were randomized among animals. The amount of time spent exploring each object (nose sniffing and head orientation within < 1.0 cm) was recorded and evaluated by the experimenter blind to the rat housing condition. Arena and objects were cleaned with 10% ethanol between trials to stop the build-up of olfactory cues. Rats were tested at P30 because this age corresponds to a maturational stage of recognition memory at which SC animals exhibit novel object recognition at 1 and 24h^61^.

### Immunohistochemistry

Animals were perfused transcardially with 4% paraformaldehyde in phosphate buffer. In order to avoid circadian effects, all animals were sacrificed during the same time interval each day (9:00–12:00 h; light phase). Brains were post-fixed and impregnated with 30% sucrose in phosphate buffered saline (PBS). Coronal brain sections (40 µm) were cut on a freezing microtome and collected in PBS before being processed for immunohistochemistry. To reduce the number of animals needed for the research, we used the same brain samples to analyze, at a given time point, all the molecular markers tested, investigating each of them within a time window consistent with its known developmental trajectory. After a blocking step, free-floating slices were incubated O/N at 4 °C in a solution of primary antibody (NeuN, Millipore, 1:500; IGF-1, IBT, 1:100; GAD67, Millipore, 1:1000; MBP, Covance, 1:500; rpS6Ser235/236, Cell Signaling Technology, 1:100; rpS6Ser240/244, Cell Signaling Technology, 1:200). Antigen-antibody interaction was revealed with either suitable Alexa Fluor-conjugated secondary antibodies (1:400, Invitrogen) or biotinylated secondary antibody (1:200, Vector Labs) followed by fluorescein-conjugated extravidin (1:300, Sigma). All sections were then mounted on gelatinized slides with Vectashield (Vector Labs).

### Immunoreactivity analysis

To compare different specimens, the parameters of acquisition (laser intensity, gain, offset) were optimized at the start and then held constant throughout image acquisition. All sections were acquired in random order in a single session to minimize fluctuation in laser output and degradation of fluorescence. **IGF1/NeuN/GAD67-** To analyze the number of IGF1, NeuN and GAD67 positive cells, sections of the binocular visual cortex were acquired at 25 × using a confocal microscope (Leica DM 6000). Collected images were imported to the image analysis system MetaMorph and counts were done on the entire Oc1B. For each animal, at least three sections were analyzed. **MBP/rpS6-** To evaluate MBP distribution in the visual cortex, images from anti-MBP sections were captured at 10 × magnification using a confocal Leica microscope and imported to MetaMorph. Measurements of mean pixel intensity in each layer of Oc1B were obtained from three measuring boxes that were randomly placed in each cortical layer. The relative MBP pixel intensity was calculated as the ratio between the mean pixel intensity of the MBP staining in each layer of Oc1B and the background signal, measured in overlying regions of subcortical white matter (WM). For each animal, at least three sections were analyzed. To evaluate pixel intensity of rpS6 Ser235/236 and rpS6Ser240/244 cellular immunofluorescence, sections of visual cortex were acquired at 25 × magnification using a confocal Leica microscope and images were imported to MetaMorph. Measurements of mean pixel intensity in the cortex were obtained from boxes randomly placed in each cortical layer. The resulting values were subtracted by mean background level, measured in overlying regions of subcortical white matter (WM). For each animal, at least three sections were analyzed. All measurements, cell counts or signal intensity, were done blind to the rearing conditions. For all antigens tested, we also performed a layer-by-layer analysis of the immunoistochemical data to check for a possible layer-specific delay of cortical maturation in animals reared in impoverished conditions.

### Maternal care observations

Postpartum maternal behavior was observed inside mating SC and IE cages, applying a standard observational protocol^90^. Behavioral observations started at postnatal day (P) 1 and were conducted every second day until P11. Maternal behavior was scored during 2 daily observation sessions of 45 min each. Observation sessions occurred at 5 p.m. and 7 p.m.; the last session was performed under dim red light, during the dark phase of the daily cycle. During each session, the behavior of each female was scored every 3 min, recording whether the target behavior was present or not. Data are reported as the percentage of observations in which pups received the target behavior. Scored behaviors (not mutually exclusive) were:

Mother in the nest (passive stimulation) — the mother is touching at least one pup with a part of the body other than the tail.

Stepping — the mother steps gently over the pups’ body and stimulates them.

Licking — general licking of any part of a pup body.

Nest building — activities aimed at nest construction and reconsolidation.

Arched back nursing — the mother is immobile and in a characteristic high upright dorsal arched posture with all or most pups attached to the nipples; this position provides the most effective nourishment for the pups.

### Determination of IGF-I concentration in maternal milk and pup serum

Milk samples were collected from P3 and P9 lactating mothers. Dams were separated from their pups between 9 and 11 AM, anaesthetized using isoflurane (3%) and subcutaneously injected with synthetic oxytocine (5 IU, Sigma). Milk was collected by manually expression from individual teats and stored at −80 °C prior to analysis. Milk samples were centrifuged at 14000 rpm at 4 °C for 30 min to separate the whey (infranatant) from the fat (supernatant) and casein (pellet). The whey milk was acid-ethanol extracted to remove IGF-I binding proteins. Blood samples were collected from P12 pups between 9 and 11 AM. Pups were anaesthetized with chloral hydrate (100 mg/kg) and blood was taken through cardiac puncture. Serum is the liquid fraction of whole blood that is collected after the blood is allowed to clot (2 h, RT). The clot was removed by centrifugation (4000 rpm, 10 min) and the resulting supernatant was carefully removed. The concentration of IGF-I was determined by radio immunoassay (RIA) using a commercial kit (Pantec s.r.l., Italy), with a sensitivity of 0.1 ng/ml.

### Statistical analysis

All statistical analyses were performed using SigmaStat Software. Differences between two groups were assessed with a two-tailed t test. The significance of factorial effects and differences among more than two groups were evaluated with ANOVA/RM ANOVA followed by Holm-Sidak test. Rank transformation was exploited for data not normally distributed. The level of significance was p < 0.05.

## Electronic supplementary material

Supplementary information
