# Author Correction: Early impoverished environment delays the maturation of cerebral cortex

**DOI:** 10.1038/s41598-021-00562-6

**Published:** 2021-12-02

**Authors:** Roberta Narducci, Laura Baroncelli, Gabriele Sansevero, Tatjana Begenisic, Concetta Prontera, Alessandro Sale, Maria Cristina Cenni, Nicoletta Berardi, Lamberto Maffei

**Affiliations:** 1 Institute of Neuroscience, National Research Council (CNR), Via Moruzzi 1, 56124 Pisa, Italy; 2 Department of Neuroscience, Psychology, Drug Research and Child Health NEUROFARBA, University of Florence, Area San Salvi – Pad. 26, 50135 Florence, Italy; 3 Fondazione G. Monasterio CNR-Regione Toscana, Via Moruzzi 1, 56124 Pisa, Italy

Correction to: *Scientific Reports* 10.1038/s41598-018-19459-y, published online 19 January 2018

The original version of this Article contained an error in Figure 7.

In Figure 7a, the image presented for the SC (Ser 235/236) panel was incorrect, as the image for the SC (Ser 240/241) panel was inadvertently included in place of the correct image. In addition, the images presented focused on different regions within layers V/VI of the visual cortex.

The original Figure 7 and accompanying legend appears below.

**Figure 7:**
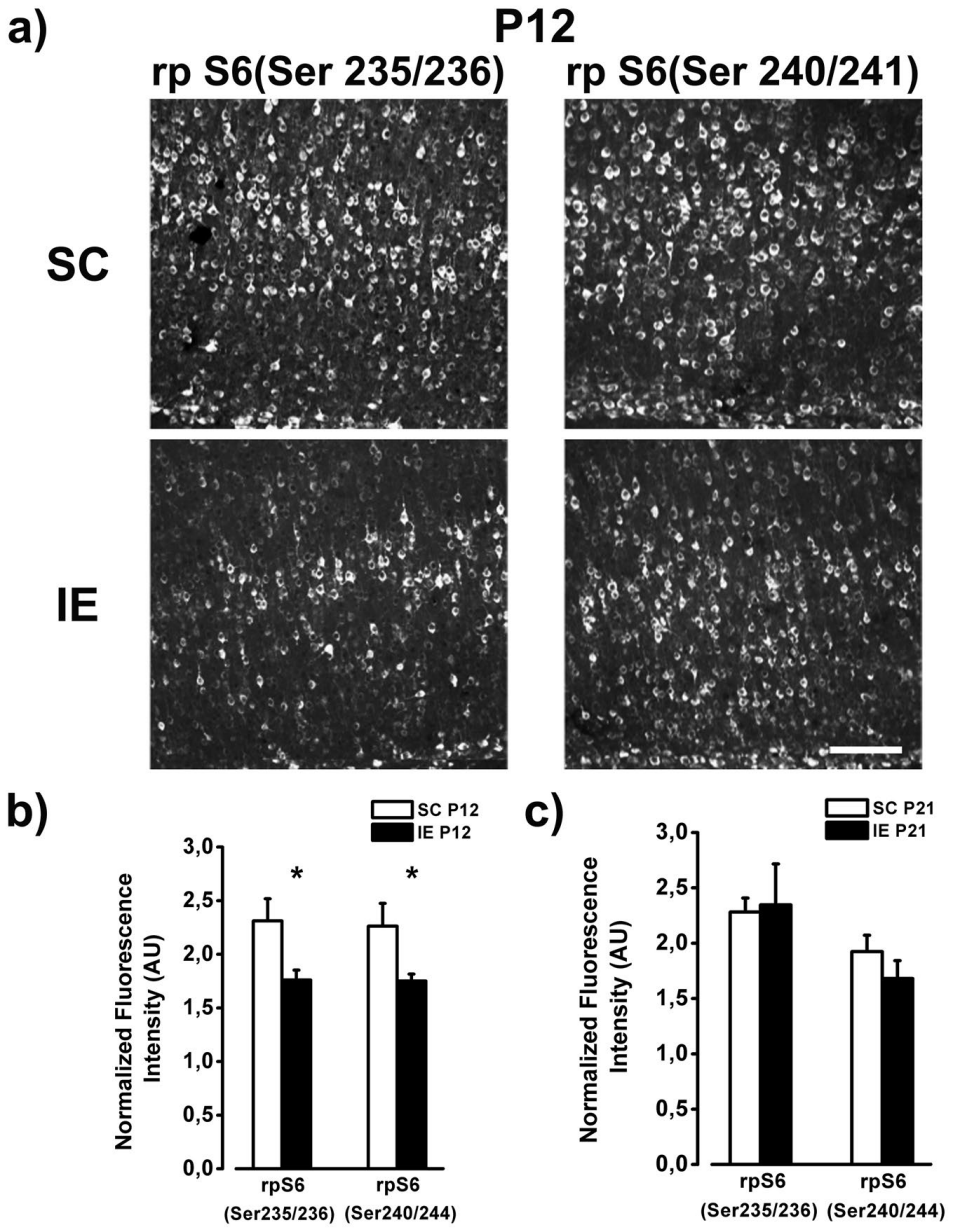
Hypophosphorylation of rpS6 in IE brain. (**a**) Example of rp S6 labeling (Ser235/236 and Ser 240/241) from fields taken in the layers V/VI of the visual cortex of P12 SC and IE rats. Calibration bar: 100 µm. (**b**) Quantitative analysis of rp S6 immunofluorescence intensity in the visual cortex of P12 animals. SC animals showed higher rpS6 expression in comparison to IE animals both for the site Ser235/236 and for the site Ser240/244 (SC P12, n = 5; IE P12, n = 5, Two way ANOVA, post-hoc Holm-Sidak method, *p* < 0.05). (**c**) At P21 rp S6 expression did not differ between SC and IE animals (SC P21, n = 5; IE P21, n = 6; *p* = 0.48). Histograms represent average values ± SEM. *p < 0.05.

The original Article has been corrected.

