# Supplementary material for: Early impoverished environment delays the maturation of cerebral cortex
Source: Sci Rep. 2018 Jan 19;8:1187. doi: 10.1038/s41598-018-19459-y (PMC5775315; doi:10.1038/s41598-018-19459-y)
Supplement: Supplementary file 1 — Supplementary information [file 41598_2018_19459_MOESM1_ESM.pdf]

Early impoverished environment delays the maturation of cerebral cortex

Roberta Narducci[1,2], Laura Baroncelli[1*], Gabriele Sansevero[1,2], Tatjana Begenisic[1], Concetta Prontera[3], Alessandro Sale[1], Maria Cristina Cenni[1], Nicoletta Berardi[1,2], Lamberto Maffei[1]

[1] Institute of Neuroscience, National Research Council (CNR), Via Moruzzi 1, I-56124 Pisa, Italy
[2] Department of Neuroscience, Psychology, Drug Research and Child Health NEUROFARBA, University of Florence, Area San Salvi – Pad. 26, I-50135 Florence, Italy
[3] Fondazione G. Monasterio CNR-Regione Toscana, via Moruzzi 1, I-56124 Pisa, Italy.

*Corresponding author:
Laura Baroncelli
Institute of Neuroscience, National Research Council (CNR)
via Moruzzi 1, Pisa I-56124, Italy.
Email: baroncelli@in.cnr.it
Tel: +390 503 153199 Fax: +390 503 153220

Supplementary figures

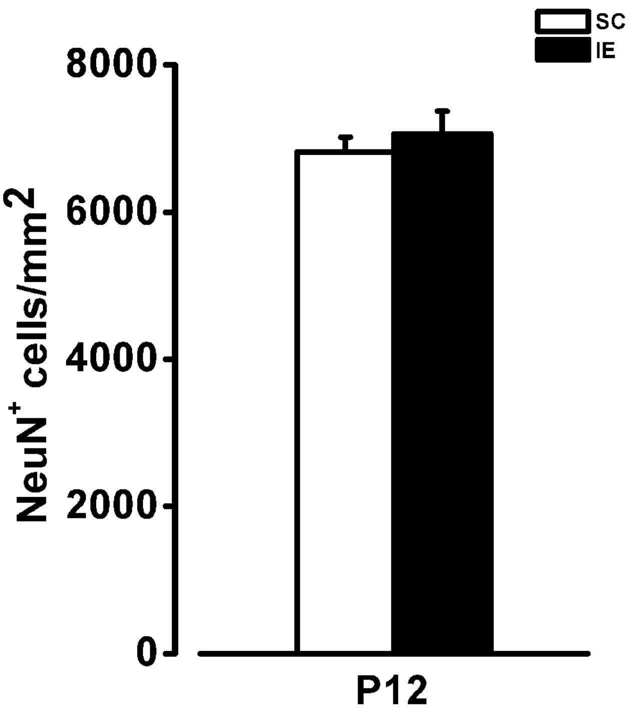

Fig. S1 Unaltered neuronal density in the visual cortex of IE animals
The number of NeuN positive cells per mm^2 in the visual cortex did not differ between SC and IE at P12 (SC, n = 6; IE, n = 6; t-test, p = 0.52). Histograms represent average values ± SEM.

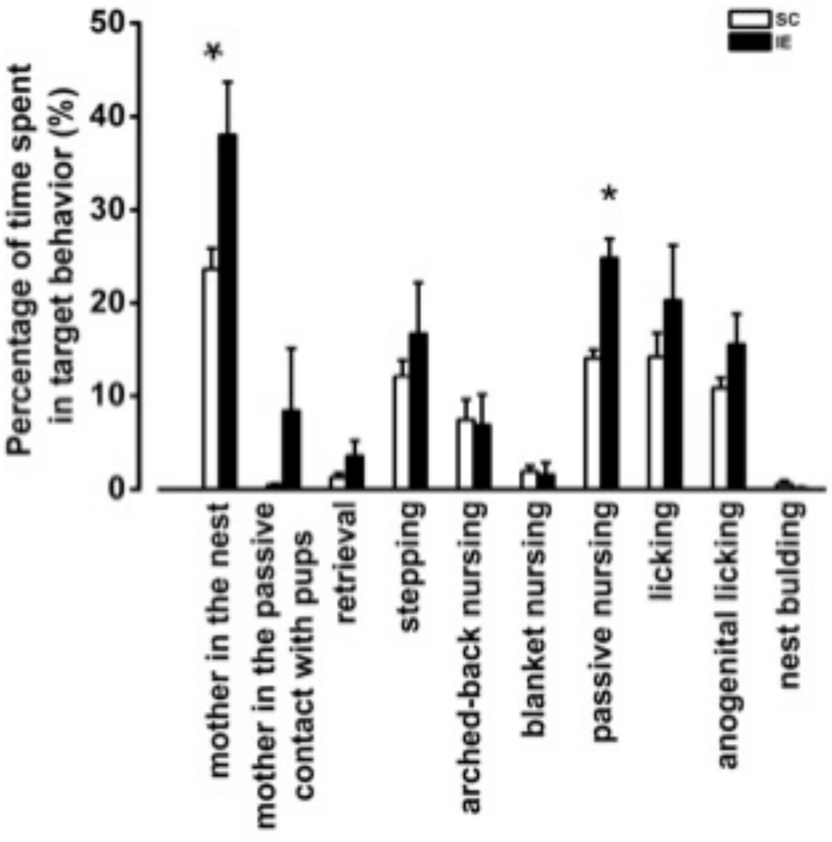

Fig. S2 Unaltered maternal care levels in IE condition

All behaviors were observed in n = 4 SC and n = 4 IE litters. Mother in passive contact with pups, retrieval, stepping, arched-back nursing, blanket nursing, licking, anogenital licking and nest building received by pups were not different between SC and IE mothers (t-test, $p = 0.284$, $p = 0.226$, $p = 0.455$, $p = 0.89$, $p = 836$, $p = 0.387$, $p = 0.22$, $p = 0.346$), while frequency of mother in the nest and passive nursing were even significantly higher in IE dams (t-test, $p < 0.05$ and $p < 0.01$, respectively). Histograms represent average values ± SEM. *, $p < 0.05$; ** $p < 0.01$.
